# Causal Relationships among Technology Acquisition, Absorptive Capacity, and Innovation Performance: Evidence from the Pharmaceutical Industry

**DOI:** 10.1371/journal.pone.0131642

**Published:** 2015-07-16

**Authors:** Jieun Jeon, Suckchul Hong, Jay Ohm, Taeyong Yang

**Affiliations:** 1 Graduate School of Innovation and Technology and Management, Korea Advanced Institute of Science and Technology, Daejeon-city, Korea; 2 Department of R&D Strategy, Korea Health Industry Development Institute, Cheongju-city, Chungcheongbuk-do, Korea; Universidad Veracruzana, MEXICO

## Abstract

This paper discusses the importance of absorptive capacity in improving a firm’s innovation performance. Specifically, we examine firm interaction with the knowledge and capabilities of outside organizations and the effect on the firm’s bottom line. We use the impulse-response function of the vector auto-regressive model to gain insight into this relationship by estimating the time required for the effect of each activity level to reach outputs, the spillover effects. We apply this methodology to pharmaceutical firms, which we classify into two sub-groups – large firms and medium and small firms – based on sales. Our results show that the impact of an activity on any other activity is delayed by three years for large firms and by one to two years for small and medium firms.

## Introduction

Since the concept of absorptive capacity was introduced by Cohen and Levinthal [1], many studies have focused on the relationships among technological acquisition, absorptive capacity, and innovation performance [1–6]. Studies have argued that firms need to acquire external knowledge to innovate and that absorptive capacity determines the magnitude of innovation performance. Many firms acquire external knowledge through various means including mergers and acquisitions (M&A) and strategic alliances, particularly in R&D-intensive sectors that emphasize technological innovation. However, there is mixed evidence concerning the effects of acquisition activity on innovation performance in the pharmaceutical industry [7–8]. This may be a result of the pharmaceutical industry’s characteristics, which differ from those of other sectors. Specifically, in the pharmaceutical industry, innovation (new drug development) requires a significant amount of time, and the innovation process is divided into several stages.

Considering these unique characteristics, this study aims to identify the causal relationships among technology acquisition, absorptive capacity, and innovation performance in the pharmaceutical industry. The detailed objectives are as follows. First, newly acquired external knowledge takes time to affect innovation performance. Therefore, we identify the time required for technology acquisition, absorptive capacity, and innovation performance to affect performance. Next, we determine how much the levels of acquisition, absorptive capacity, and innovation performance affect each other—that is, the degree of the interaction among them. For example, if the level of acquisition increases, to what extent does the impulse response of absorptive capacity or innovation performance increase or decrease?

We classify pharmaceutical firms into two groups depending on the size of the company—large pharmaceutical firms and small and medium pharmaceutical firms. In large firms, a one-unit shock of acquisition on absorptive capacity (R&D intensity) appears after three years, and the effects gradually disappear after three years. A one-unit shock of acquisition also increases innovation performance (the difference in the number of patents granted between a prior year and a subsequent year; we used the first differentiated patent variable as a measure of innovation performance in large pharmaceutical firms for statistical analysis. For more information, refer to the results section (section 4).) and peaks after three years. A one-unit shock of R&D intensity affects acquisition and innovation performance after three years. In small and medium firms, the unit shock of acquisition on patents granted increases over time, and that the unit shock of R&D intensity on acquisition and innovation appears to have effects in the current year and one subsequent year. This implies that high absorptive capacity affects acquisition activity and innovation performance within a short time.

This study has three main contributions. First, we present the effect of acquisition on innovation performance quantitatively by considering absorptive capacity as a mediator. This develops the technology acquisition and absorptive capacity theory quantitatively. Second, we determine the time required to impact innovation performance after external technical knowledge is acquired. We suggest that small and medium firms achieve innovation performance quickly after acquiring external technical knowledge; achieving innovation performance takes longer for large firms. Previous studies have argued whether technology acquisition strategy leads to positive innovation performance. This study shows that it takes time to create innovation performance. We cannot assume immediate positive or negative performance effects. Third, this study contributes to the existing literature on economics and finance in terms of the methodology employed. Our methodology demonstrates the interaction among variables quantitatively with time lag. We use a novel methodology of panel vector-autoregressive model for this study of innovation.

The remainder of the paper is organized as follows: Section 2 provides technological sourcing in the pharmaceutical industry. Section 3 presents the theoretical development and hypotheses. Section 4 explains the data and our empirical specifications. Section 5 discusses the results of the analysis, and Section 6 presents the conclusions and implications of this study.

## Technological Sourcing in the Pharmaceutical Industry

Technological sourcing occurs in high technology industries. Various types of technological sourcing activities exist such as co-research, research contracts, license, marketing agreement, joint venture, and M&A. Among them, acquisitions of firms are common method of gaining technology capacity in high technology industries [9]. Such technological sourcing activities are particularly prevalent in the pharmaceutical industry and have been increasing since 1980s with a rapid increase noted in the 2000s [10]. Whittaker and Bower [11] predicted that the phenomenon would be becoming frequent. In the context of the increasing external technology sourcing in the pharmaceutical industry, we consider the motivations of technological sourcing from three aspects. First, the characteristics of pharmaceutical industry include substantial time requirements for a new drug development and substantial investment. Gassmann et al. [12] estimated that pharmaceutical firms require, on average, 13.2 years to bring new drugs to market. DiMasi et al. [13] found that the firms typically invest $800 million for development of new drug. These characteristics differentiate the pharmaceutical industry from other industries and may hinder that pharmaceutical firms’ performance in the development of a new drug. Therefore, these risk factors cause pharmaceutical firms to undertake technological sourcing activities to reduce the time required for product development and the associated costs. Second, R&D productivity is reduced or stagnant. R&D productivity is defined as the number of new drugs per funded R&D cost for a unit. Kim [14] noted that while firms’ R&D investment costs increase continuously, the number of new drugs approved from the Food and Drug Administration (FDA) is decreasing. The author estimated that the number of new drugs per US $10 billion R&D cost decreased from 3.13 to 0.63 between 1996 and 2003. This encourages pharmaceutical companies to engage in technological acquisition activity. Lastly, looming patent expiration dates of blockbuster drugs are a concern. After patent expires, pharmaceutical firms may be faced with declining sales because generic drug-focused firms introduce generic drugs just after expiration date of patent. This activity causes a decline in sales of original drugs of up to 60% for pharmaceutical firms [14]. This problem may be a threat to firms’ financial capability and is exacerbated in cases where firms’ total sales depend on blockbuster drugs. For these reasons, pharmaceutical companies must participate in technological sourcing activities to acquire new technology and know-how to expand and diversify their product pipeline and reduce risk.

## Theoretical Development

Chesbrough [15] asserted that, in recent years, using internal resources to increase knowledge exchange and employee flexibility has proved to be an inefficient strategy, and the popularity of the Internet, the formation of the venture capital market, and active external suppliers has spurred wide cooperation in technological innovation. Activities to acquire and exploit knowledge developed by others can facilitate development capability and minimize a firm’s concerns regarding technological uncertainties [16]. The main reason that firms attempt to acquire external knowledge is to gain technical know-how and develop technical capabilities [17]. Rosenberg [18] insisted that knowledge from external sources is a pivotal element in the success of a firm’s innovation. Previous studies have focused on firms’ acquisitions to meet needs, which may include technical knowledge or financial capacity [19–20]. Higgins and Rodriguez [8] examined the performance of 160 pharmaceutical acquisitions from 1994 to 2001 and found that, on average, acquirers realized significant positive returns. Therefore, in this study, we define external technological acquisition as a firm obtaining technical or scientific knowledge from outside sources such as R&D programs of other subjects. Further, in our definition, external knowledge acquisition includes intellectual property like patents and technical know-how. Thus, this study differs from previous research focused on firms’ acquisitions.

Additionally, many previous studies have focused on the role of absorptive capacity. More recently, the relationships among acquisition, absorptive capacity, and innovation performance have been the focus of some researchers. Many researchers have found that a firm’s innovation depends on its absorptive capacity after acquiring external knowledge [1–6], beginning with Cohen and Levinthal’s [1] assertion that absorptive capacity, defined as a “firm’s ability to recognize the value of new, external knowledge, assimilate it, and apply it to commercial ends,” ([1] p. 128) contributes to creating outputs. Cohen and Levinthal [1] introduced absorptive capacity as the capabilities to recognize and assimilate the value of new knowledge and apply it to commercial purposes. Mowery and Oxley [21] defined absorptive capacity as a broad set of skills needed for the tacit component of transferred knowledge and the need to modify this imported knowledge. Kim [22] said that absorptive capacity is a learning capability and includes the problem-solving skills that enable a firm to assimilate knowledge and create new knowledge. Later, several studies suggested a comprehensive definition of absorptive capacity by constructing a multidimensional construct of abilities [23–25]. For this study, we use the definition of absorptive capacity proposed by Cohen and Levinthal [1]. Stuart and Podolny [26] suggested that learning, as an activity that involves an accumulation process is maximized when the area of accumulated knowledge is the focus, and Sørensen and Stuart [3] stated that firms may have difficulty in assimilating knowledge if they have a weak absorptive capacity. Teece [6] also showed that the accumulation of external knowledge improves a firms’ likelihood of recognizing and developing a new technological opportunity. Several studies have suggested that absorptive capacity functions as a mediator between knowledge acquisition and innovation [27–30]. Van den Bosch et al. [27] concluded that absorptive capacity functions as a mediator to create new knowledge. Darroch and McNaughton [29] showed that knowledge acquisition affects innovation indirectly. Lane et al. [28] asserted that a recursive relationship exists among knowledge acquisition, absorptive capacity, and innovation. Escribano et al. [30] examined the relationships among external knowledge management, absorptive capacity, and innovation to show that the absorptive capacity of a firm mediates between external knowledge and innovative outcomes. Liao et al. [31] also found that absorptive capacity is a mediator between knowledge acquisition and innovation capability in the finance and manufacturing industry.

However, we need to have a better grasp of the relationships between each of these components and the causal relationship among them. In this study, by considering the relationships among knowledge acquisition, absorptive capacity, and innovation, we focus on identifying the dynamic interactions of these components and the time it takes to cause an effect on each if an activity increases with a one-unit shock. Thus, this study mainly aims to identify the time required to create absorptive capacity and innovation performance after acquiring external technical knowledge.

Research on the relationship between absorptive capacity and firm performance considered firm age and size as moderating factors for small and medium firms [32]. For small and medium firms, absorptive capacity is significant because the capacity depends on the firm’s ability to effectively and rapidly develop and expand the organizational knowledge base [32, 33]. For example, constant interaction with markets, stock markets, entrepreneurship, and social networks may affect the relationship between absorptive capacity and firm performance for small and medium sized firms. However, we attempt to capture the effects of external technology acquisition on innovation performance by considering the absorptive capacity of firms. We focus on the recursive relationships between absorptive capacity and innovation [28] to obtain a solid grasp of the relationships between each of these components and the causal relationship among them. This study examines the relationships among knowledge acquisition, absorptive capacity, and innovation identifying the dynamic interactions of these components and the time required for an increase of a one-unit shock to cause an effect on each component. Thus, this study identifies the time required to create absorptive capacity and innovation performance after acquiring external technical knowledge.

## Empirical Strategy

### Data

We used several data sources to conduct the analysis. First, Datamonitor’s Medtrack [34] provided critical data about technology acquisitions by pharmaceutical firms. Second, Compustat [35] provided data related to the R&D intensity of pharmaceutical firms. Finally, Wintelips [36] provided the number of patents granted to pharmaceutical firms. All data are from the US. The data construction can be viewed as unbalanced panel data. We used 98 pharmaceutical companies (S1 Appendix). Because not all companies existed during the entire period, we used a sample of firms active from the year 1990 to the year 2011 even if they did not conduct operations during the entire period. The period covered is from the year 1990 to the year 2011 because the innovation process of the pharmaceutical industry typically requires over 15 years. The US pharmaceutical industry is more dynamic than that of other countries in terms of acquisitions, R&D intensity, and patent filings; therefore, we selected US-based pharmaceutical firms for this study by focusing on SIC codes 2834, 2835, and 2836.

The data contain the number of external technology acquisitions, R&D intensity, and the number of patents granted. Acquisition is defined as an important means of bringing external knowledge into the firm [37]. We measure acquisition by counting the number of technological acquisitions. Prabhu et al. [38] counted the number of acquisition activities to examine the effect of innovation acquisition on innovation for the pharmaceutical industry. For this research, data on the number of external technical knowledge acquisitions from 1990 to 2011 were gathered from Datamonitor’s Medtrack [34]. The data represent how many pharmaceutical firms acquired technologies or technical know-how from other companies through R&D programs. In previous research, R&D intensity was used to measure the performance of absorptive capacity. For example, Cohen and Levinthal [1] suggested that R&D intensity would be the most appropriate measure of the performance of absorptive capacity, because the firm’s internal organization must constantly find a way to efficiently absorb its external R&D investments. R&D intensity can be calculated by dividing the R&D expenditure by the total sales as obtained from Compustat [35]. Although most data points are populated with reliable values, there are several data points with missing values for R&D expenditure or total sales. We eliminate the data points with missing values giving 301 pharmaceutical companies in total (SIC code 2834, 2835, 2836). We also exclude companies with missing values in either R&D expenditure or total sales or both and companies with non-availability for gathering patent counts. Innovation performance can be measured in several ways. Prior research used patent counts [39–43] and patent citations [2, 44–47]. This study uses annual patent count as a proxy for innovation performance because patents embody technical knowledge. We collect the annual number of patents granted from the year 1990 to the year 2011 as provided by Wintelips [36]. Table 1 shows the measurements and sources of variables used for this study.

**Table 1:** Measurements and sources of variables.

Variable	Measurement	Source
External technology acquisition	The count of R&D programs or projects from other firms	Medtrack
Absorptive capacity	The annual R&D intensity (R&D expenditure/Total sales)	Compustat
Innovation performance	The annual count of paten newly granted	Wintelips

AC, Acquisition of external technology; RD, Absorptive capacity (R&D intensity); PT, Innovation performance (patents granted).

This study covers 98 pharmaceutical companies. The data structure is based on unbalanced panel data including cross sectional and time series forms. We target only US-based global and public firms only that have engaged in a significant amount of innovation-related activities over the last two decades. We analyze technology acquisition, R&D intensity, and patent count to understand the effect of those factors on innovation performance. We classify firms into two groups based on sales: large firms (sales greater than $100 million) and small and medium firms (sales less than $100 million). In total, 29 pharmaceutical firms are assigned to the large class and the other 69 firms are assigned to the small and medium class. Table 2 presents the summary statistics for main variables.

**Table 2:** Summary statistics for main variables.

Firm’s group	Variables	Mean	Maximum	Minimum	Standard deviation
**Large pharmaceutical firms**	**AC**	1.263	9.000	0.000	1.625
	**RD**	24.580	951.093	0.823	67.626
	**DPT**	3.241	260.000	-269.000	36.547
**Small and medium pharmaceutical firms**	**AC**	0.340	5.000	0.000	0.688
	**RD**	1661.144	444500.000	0.000	17053.71
	**PT**	5.689	109.000	0.000	12.547

AC, Acquisition of external technology; RD, Absorptive capacity (R&D intensity); PT, Innovation performance (patents granted), DPT (first-differentiated variable of patents granted).

### Model Specification

This study examines the relationships among technology acquisition, absorptive capacity, and innovation performance using the impulse-response function (IRF) of the vector auto-regressive model (VAR). VAR has been used to forecast several variables [48–49]. We extend the univariate autoregressive model to multiple time-series variables, also known as the vector of time-series variables. If the lag period in each equation is identical to *p*, the equation takes the form of VAR(*p*), which is expressed as follows:

$$Z_{it}=\Gamma_{0}+\Gamma_{1}Z_{it-1}+\cdots +\Gamma_{p}Z_{it-p}+f_{i}+\varepsilon_{t}$$ (1)

where *Z*_*it*_ stands for a three-variable vector (acquisition of external technical knowledge, R&D intensity as a proxy for absorptive capacity, patents granted as an indicator for innovation performance), *f*_*t*_ stands for fixed effects, Γ_*k*_ stands for estimated scalar value for k-period time lag, and *ε* stands for an error term.

Previous studies on the relationships among knowledge acquisition, absorptive capacity, and innovation considered time lag [19, 50]. Cincera [50] examined the time pattern of the lag between R&D investment and technological spillover on patenting activity. Ahuja and Katila [19] examined the relationship between (non)technological acquisition and innovation performance and considered the time lag between them. The authors examined the effect of technological acquisitions on subsequent innovation performance in the chemical industry. The authors also considered lagged independent variables with a time lag of four, the number of non-technological acquisitions, absolute size of acquired knowledge base, relative size of acquired knowledge base, relatedness of acquired knowledge base, and the number of technological acquisitions as independent variables in the panel regressive model. Desyllas and Hughes [46] also estimated the acquisition effect of the three post-acquisition years. Based on this, we consider a time lag of three to four as the optimal time lag. We tested to determine the optimal time lag of VAR from one to five repeatedly. It is important to determine the optimal time lag of VAR because impulse may depend critically on the lag order of the VAR model fitted to the data. These differences can be large enough to affect the substantive interpretation of VAR impulse-response estimates [51, 52]. Therefore, first, all possible time lags are based on previous studies [19, 50, 53]. Second, we tested five models with possible time lags of one to five to find the optimal time lag. The results showed that more significant variables were shown in VAR (3) than VAR (1) and VAR (2). Different results were not shown for a time lag of four and five compared with a time lag of three. Significant variables were shown more in VAR (3) than the other models; therefore, we use a time lag of three (*p*) for our analysis.

This study adopts the panel VAR methodology used by Love and Zicchino [54]. This methodology allows us to analyze the complex interactions among variables. To apply VAR to panel data, the data structure must be the same for each cross-sectional unit. However, this restriction cannot be satisfied with real data. We use the fixed effects, denoted by *f*_*i*_, to solve the problem by allowing individual heterogeneity in the variable levels. In the fixed-effects model, the lagged dependent variable is included in regressors; thus, the endogeneity may occur because error terms can be correlated with regressors. This would bias the estimation of coefficients. To circumvent this problem, various estimating methods have been suggested using instrument variables [55–57]. We follow the Helmert procedure [58] to eliminate the problem associated with biased estimation. Arellono and Bover [58] suggested the generalized method of moments (GMM) system for removing fixed effects and preserving the orthogonality between transformed variables and lagged regressors.

An IRF represents the reaction of a variable to a one-unit shock of another variable. In this study, we calculated the standard errors of IRFs and created confidence intervals with Monte Carlo simulations. We repeated the experiment 1,000 times and generated 5^th^ and 95^th^ percentile distributions as a confidence interval for the impulse responses. We experimented with a several repetitions and obtained similar results.

The main purpose of this study is to identify how much time is required for external knowledge acquisition to affect absorptive capacity and innovation performance. We use the IRF of panel VAR; the output is represented by a brief input signal called an impulse. An impulse response refers to the reaction of any dynamic system to some external change. Bottazzi and Peri [59] used the IRF to determine the impulse response of the level of R&D and the stock of knowledge in the US and other countries. The IRF is used to analyze the dynamic effects of the system when the model receives the impulse. We use Stata to analyze the IRF, a method developed by Love and Zicchino [54].

### Empirical Results

To analyze impulse response, we first conduct a unit root test for a variable’s stationarity. For large pharmaceutical firms, the first two variables—the number of technology acquisitions and R&D intensity—are stationary, but the third variable—patents granted—is non-stationary (Tables A and B in S2 Appendix). Therefore, we introduced the first-differentiated patent variable and identified it as stationary when first differentiated. The result implies that the variable, PT, should be substituted by the difference of PT between the current year and the prior year. The variables is considered as the increase in the number of patents newly granted between year t and year t-1. For small and medium pharmaceutical firms, all variables are stationary. We used Stata to estimate the GMM and impulse responses of Love and Zicchino [54]. We estimate GMM using a time lag of three. Table 3 shows the results of the model for large firms and small/medium pharmaceutical firms.

**Table 3:** The results of a 3-variable VAR model with time lag of three.

		Dependent variable		
Firm’s group	Independent variable	AC(t)	RD(t)	DPT(t)(or PT(t))
**Large pharmaceutical firms**	**AC(t-1)**	0,478(6.322)*	-0.611(-1.529)***	1.241(0.486)
	**AC(t-2)**	0.085(1.197)	-0.370(-0.077)	-3.323(-1.873)***
	**AC(t-3)**	0.252(3.819)***	1.142(1.590)***	4.393(2.027)*
	**RD(t-1)**	-0.000(-0.072)	0.421(1.169)	-0.004(-0.412)
	**RD(t-2)**	-0.001(-1.605)	0.272(1.251)	-0.003(-0.653)
	**RD(t-3)**	0.002(7.633)*	1.142(1.590)	0.011(1.850)***
	**DPT(t-1)**	-0.001(-0.666)	-0.004(-0.673)	-0.051(-0.402)
	**DPT(t-2)**	-0.005(-2.459)	-0.001(-0.130)	0.124(1.296)
	**DPT(t-3)**	-0.006(-2.922)	-0.009(-1.376)	-0.070(-0.870)
**Small and medium pharmaceutical firms**	**AC(t-1)**	0.133(2.064)	-137.331(-0.349)	0.580(1.014)
	**AC(t-2)**	0.185(3.113)***	-262.661(-0.853)	0.493(1.106)
	**AC(t-3)**	0.091(1.888)	-366.042(-0.946)	0.682(1.410)
	**RD(t-1)**	7.98e-07(0.934)	0.500(1.115)	8.37e-07(0.240)
	**RD(t-2)**	8.72e-07(1.387)***	-0.291(-1.164)	-3.69e-06(-1.267)
	**RD(t-3)**	-1.97e-06(-1.628)***	0.130(0.807)	2.37e-06(0.947)
	**PT(t-1)**	-0.004(-0.496)	-25.165(-0.700)	0.875(6.093)*
	**PT(t-2)**	0.000(0.036)	46.576(0.823)	-0.010(-0.083)
	**PT(t-3)**	0.009(1.435)	-24.734(-0.681)	0.072(0.761)

AC, Acquisition of external knowledge; RD, R&D intensity; PT, patents granted; DPT, first-differentiated PT variable.

VAR model is estimated by GMM. Reported numbers show the coefficients of regressing the column variables on lags of the row variables. t-statistics are in parentheses.

* indicates significance at the 1% level.

*** indicates significance at the 10% level.

The results for large pharmaceutical firms show that AC, RD, and DPT, are most exogenous in that order based on GMM with a time lag of three. We use this order to calculate impulse responses. For small and medium firms, GMM with a time lag of three shows that PT, RD, and AC represent the order of exogenous impact, and we use this for the IRF. The IRF results depend on the order of variables. The order means the degree of exogeneity, if variable x appears earlier in the system than variable y, then x is weakly exogenous with respect to y. In this study, we change the order of variables to calculate IRFs, which represents similar results for any order.

**Fig 1:**
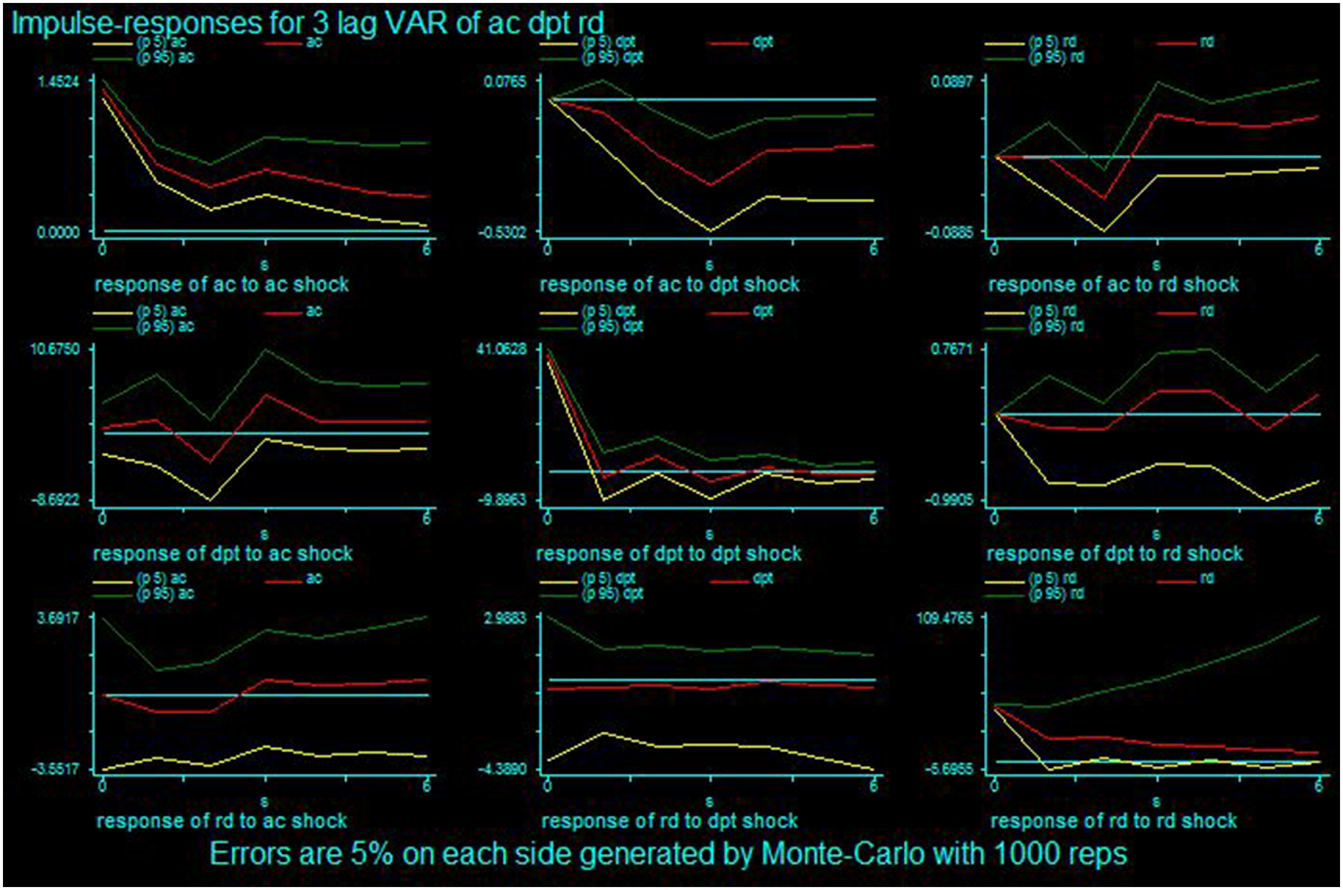
Impulse response results for the large pharmaceutical firms.

**Fig 2:**
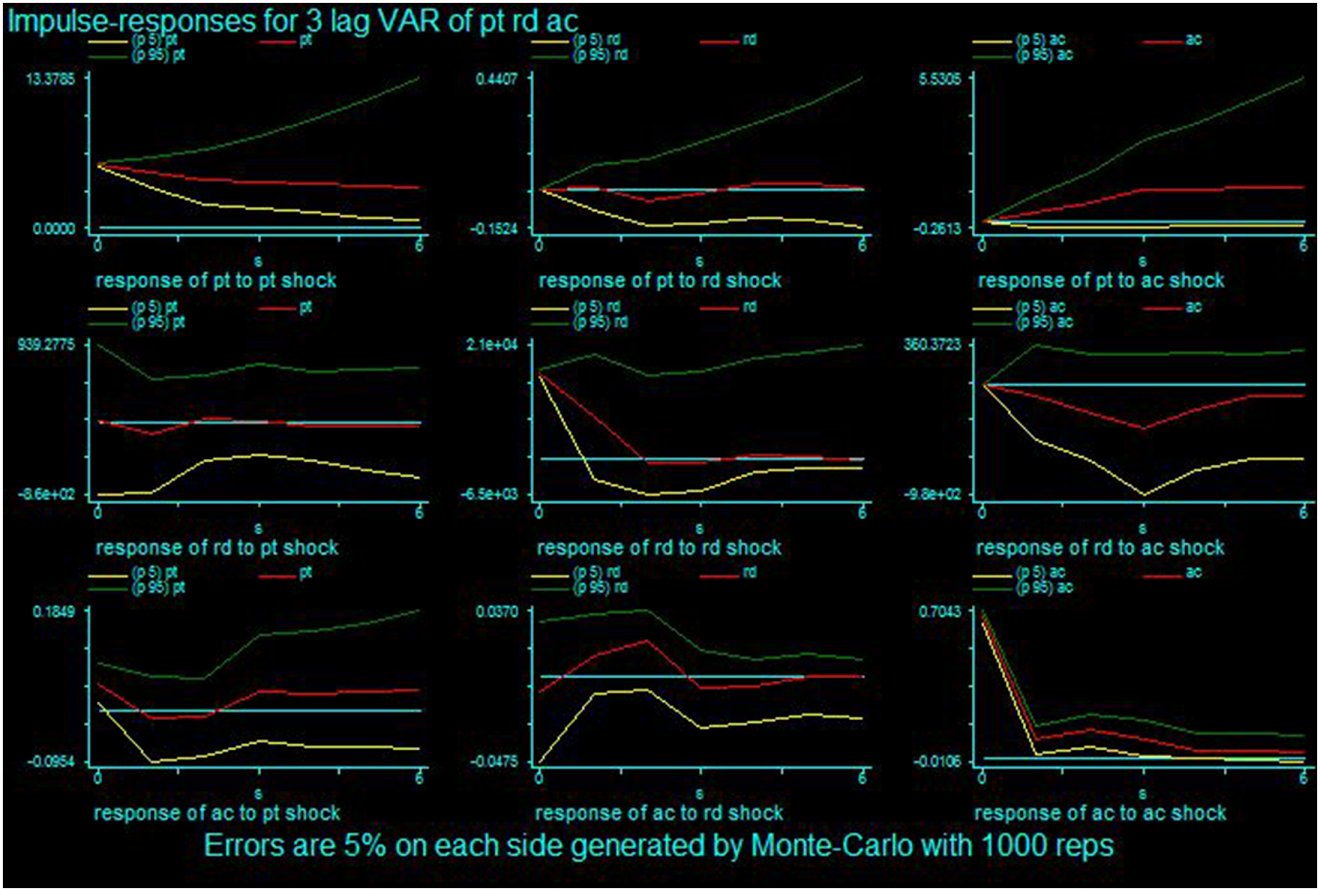
Impulse response results for the small and medium pharmaceutical firms.

The impulse responses show how long the output takes and lasts if a one-unit shock of standard deviation occurs. For this study, impulse responses indicate how long R&D intensity (or patent generation) takes to create performance and how long the effect lasts if there is a one-unit increase in technology acquisition (or R&D intensity, patent generation). In large pharmaceutical firms, the impact peaks after three years for a one-unit shock for all variables at time 0. Specifically, RD peaks after three years and DPT increases as time passes after a one-unit shock of AC. A shock to RD causes both AC and DPT to significantly react in three and four years. A shock to DPT appears in RD reacting after four years but causes AC to decrease. Table 4 shows the impulse responses for large pharmaceutical firms, and Fig 1 shows the graphs for this information. In small and medium pharmaceutical firms, the reaction appears quickly. The effect of a one-unit shock of AC on PT increases after time zero. The reaction of PT and AC to an RD shock is high in the current year and first subsequent year, but the impact is not great. The shock of PT on RD creates a substantial reaction at time zero, but AC is not affected significantly. Table 5 shows the impulse responses for small and medium pharmaceutical firms, and Fig 2 shows the graphs for this information. Overall, if a firm increases its R&D intensity by one unit, the number of newly granted patents and external technology acquisitions will increase within a short time.

**Table 4:** Impulse response results for the large pharmaceutical companies.

Variable	Time	AC	RD	DPT
**AC**	**0**	1.369	0.000	0.000
	**1**	0.654	-0.001	-0.053
	**2**	0.423	-0.046	-0.217
	**3**	0.595	0.055	-0.337
	**4**	0.4861	0.041	-0.202
	**5**	0.384	0.039	-0.194
	**6**	0.339	0.050	-0.179
**RD**	**0**	-0.043	41.062	0.000
	**1**	-0.857	17.286	-0.168
	**2**	-0.830	18.442	-0.069
	**3**	0.706	12.841	-0.309
	**4**	0.400	10.531	0.031
	**5**	0.540	8.00	-0.228
	**6**	0.720	6.373	-0.319
**DPT**	**0**	0.717	-0.500	38.921
	**1**	1.663	-0.129	-1.995
	**2**	-3.732	-0.242	4.873
	**3**	4.720	0.308	-3.323
	**4**	1.373	0.266	0.983
	**5**	1.248	-0.167	-0.884
	**6**	1.256	0.253	-0.659

AC, Acquisition of external knowledge; RD, R&D intensity (R&D expenditure divided by total sales); DPT. First-differentiated variable of patent variable.

**Table 5:** Impulse response results for the small pharmaceutical companies.

Variable	Time	PT	RD	AC
**PT**	**0**	5.647	0.000	0.000
	**1**	4.970	0.008	0.393
	**2**	4.309	-0.041	0.731
	**3**	4.148	-0.017	1.221
	**4**	3.948	0.021	1.272
	**5**	3.751	0.024	1.318
	**6**	3.601	0.009	1.335
**RD**	**0**	33.674	1.6e+04	0.000
	**1**	-1.3e+02	7.9e+03	-93.165
	**2**	51.300	-6.5e+02	-2.5e+02
	**3**	39.713	-5.7e+02	-3.9e+02
	**4**	-34.411	920.950	-2.2e+02
	**5**	-43.647	535.270	-1.0e+02
	**6**	-45.059	-70.197	-92.295
**AC**	**0**	0.050	-0.008	0.679
	**1**	-0.017	0.012	0.090
	**2**	-0.012	0.020	0.136
	**3**	0.039	-0.006	0.093
	**4**	0.031	-0.005	0.044
	**5**	0.034	0.000	0.037
	**6**	0.037	0.000	0.028

AC, Acquisition of external knowledge; RD, R&D intensity (R&D expenditure divided by total sales); PT, Patent count.

## Conclusion

This study identified the impact of external technology acquisition, absorptive capacity, and innovation performance in the pharmaceutical industry. We used panel VAR, or, more precisely, IRFs. We classified pharmaceutical firms into two classes based on sales: large firms and small and medium firms.

Our analysis shows several interesting results. First, external technology acquisition creates positive innovation performance. Our model estimates that the effect will be delayed by three years for large pharmaceutical firms and by one to two years for small and medium pharmaceutical firms. This suggests that technology acquisition takes longer to effect large firms than for small and medium firms. Second, our analysis indicates that the impact of absorptive capacity on acquisition and newly granted patents seems to occur more quickly for small and medium firms. This means that small and medium firms can obtain this effect in a relatively short time. Absorptive capacity plays an important role in external technology acquisition. According to Cohen and Levinthal [1], absorptive capacity allows firms to learn different ways to create new knowledge through the acquisition of external knowledge. Absorptive capacity is an important part of firm’s ability to acquire external knowledge. Therefore, absorptive capacity can be increased more rapidly in small and medium firms compared to large firms. This absorptive capacity quickly influences external technology acquisition activity and innovation performance because firms can develop an educational process to effectively identify, assimilate, and exploit acquired knowledge. Finally, increasing the number of acquisitions benefits innovation; the results show that a one-unit increase of acquisitions raises the count of patents granted over time. These results illustrate to managers how external knowledge creates innovation performance. Quantitative results allow managers to make decisions. The results contribute to the development of technical acquisition and absorptive capacity theory by showing that creating innovation performance requires time, and absorptive capacity leads to innovation performance within a short time.

This study has some limitations. First, further research would improve measurement for absorptive capacity. This paper used R&D intensity as an index for absorptive capacity. However, pharmaceutical firms are faced with minimum development costs for new drugs regardless of their economic potential. In this case, R&D intensity can exceed the 100^th^ percentile, and may even reach the 1000^th^ percentile. The development of a more appropriate index for measuring absorptive capacity for pharmaceutical firms is required. Next, we considered three variables—technical acquisition, R&D intensity, and patents granted—to examine the effect of technical acquisition on performance. To be precise, we should include more indexes for each factor. We classified the firms as two groups; small and medium and large firms. However, the effective factor could be different depending on the size of the firm, the firm’s capability, and environment. A complete, theoretical picture requires more factors that should capture the effect on the firm’s innovation performance depending on the firm’s size. Small and medium firms with constant interaction with markets, stock markets, entrepreneurship, and social networks possess an advantage in developing and expanding a firm’s knowledge base quickly. Therefore, a study that considers the characteristics and environmental conditions of firms should be conducted in the future. Third, there are limitations in the data. This study focused on US firms. Even though the US pharmaceutical market is highly dynamic, it may not represent the general pharmaceutical market. The comparison or inclusion of other regions such as Europe and Asia is required for generalization. Other regions have a more static pharmaceutical industry, and an extended study would provide insight to the overall structure of pharmaceutical systems. Therefore, a comparison among Asian, European, and US firms would provide insight to the overall structure of the pharmaceutical industry.

## Supporting Information

S1 Appendix Company list for analysis. (DOCX)

S2 Appendix Unit root test. This file also contains Table A and Table B. Table A, Unit root test in large pharmaceutical firms. Table B, Unit root test in small and medium pharmaceutical firms.
(DOCX)
