# Supplementary material for: Causal Relationships among Technology Acquisition, Absorptive Capacity, and Innovation Performance: Evidence from the Pharmaceutical Industry
Source: PLoS One. 2015 Jul 16;10(7):e0131642. doi: 10.1371/journal.pone.0131642 (PMC4504511; doi:10.1371/journal.pone.0131642)
Supplement: S1 Appendix — (DOCX) [file pone.0131642.s001.docx]

**S1 Appendix. Company list for analysis**

| **ID** | **Company** | **ID** | **Company** | **ID** | **Company** |
| --- | --- | --- | --- | --- | --- |
| **1** | Alpharma Inc | **34** | TARO Pharmaceutical INDS LTD | **67** | Imclone Systems Inc |
| **2** | Abbott Laboratories | **35** | Salix Pharmaceuticals LTD | **68** | Cangene Corp |
| **3** | ALZA Corp | **36** | Entremed Inc | **69** | Gilead Sciences Inc |
| **4** | WYETH | **37** | LABOPHARM Inc | **70** | Corvas International Inc |
| **5** | Bausch & Lomb Inc | **38** | Cubist Pharmaceuticals | **71** | Cell Genesys Inc |
| **6** | Bristol-Myers Squibb Co | **39** | Depomed Inc | **72** | Vical Inc |
| **7** | Chattem Inc | **40** | Eisai Co LTD | **73** | GTC Biotherapeutics Inc |
| **8** | Chiron Corp | **41** | Novartis AG | **74** | Encysive Pharmaceuticals Inc |
| **9** | Elan Corp PLC | **42** | Skyepharma PLC | **75** | NPS Phamaceuticals Inc |
| **10** | Johnson & Johnson | **43** | Penwest Pharmaceuticals Co | **76** | Novavax Inc |
| **11** | Lilly (ELI) & Co | **44** | Isotechnica Pharma Inc | **77** | Alexion Pharmaceuticals Inc |
| **12** | Merck & Co | **45** | United Therapeutics Corp | **78** | Neurocrine Biosciences Inc |
| **13** | Pharmacia Corp | **46** | Inspire Pharmaceuticals Inc | **79** | Forest Laboratories |
| **14** | Mylan Inc | **47** | Introgen Therapeutics Inc | **80** | Genentech Inc |
| **15** | Novo Nordisk A/S | **48** | Hospira Inc | **81** | Glaxosmithkline PLC |
| **16** | Pfizer Inc | **49** | Theravance Inc | **82** | Astrazeneca PLC |
| **17** | Valeant Pharmaceuticals | **50** | E-Z-EM Inc | **83** | Insite Vision Inc |
| **18** | Pharmacia & Upjohn Inc | **51** | OSI Pharmaceuticals Inc | **84** | Bioniche Life Sciences Inc |
| **19** | Warner-Lambert Co | **52** | Cytogen Corp | **85** | Valeant Pharmaceuticals INTL |
| **20** | Celgene Corp | **53** | Human Genome Sciences Inc | **86** | Geron Corp |
| **21** | Smithkline Beecham PLC | **54** | Myriad Genetics Inc | **87** | Arqule Inc |
| **22** | IVAX Corp | **55** | Millennium Pharmaceuticals | **88** | Corixa Corp |
| **23** | Columbia Laboratories Inc | **56** | Biosite Inc | **89** | Curagen Corp |
| **24** | Atrix Laboratories Inc | **57** | Heska Corp | **90** | DYAX Corp |
| **25** | Cephalon Inc | **58** | Lexicon Pharmaceuticals Inc | **91** | Life Technologies Corp |
| **26** | ISIS Pharmaceuticals Inc | **59** | Amgen Inc | **92** | Exelixis Inc |
| **27** | Cambridge Neuroscience Inc | **60** | Savient Pharmaceuticals Inc | **93** | Insmed Inc |
| **28** | Sepracor Inc | **61** | Centocor Inc | **94** | Dendreon Corp |
| **29** | Epimmune Inc | **62** | Enzon Pharmaceuticals Inc | **95** | Seattle Genetics Inc |
| **30** | Perrigo Co | **63** | Immunex Corp | **96** | Dynavax Technologies Corp |
| **31** | Ligand Pharmaceutical Inc | **64** | Repligen Corp | **97** | Methylgene Inc |
| **32** | Watson Phamaceuticals Intl | **65** | Genzyme Corp | **98** | Grifols SA |
| **33** | CIMA LABS Inc | **66** | Biogen Idec Inc |  |  |

The companies that acquired external technology from the year 1990 to the year 2011 provided by Datamonitor’s Medtrack.
