# Supplementary material for: Causal Relationships among Technology Acquisition, Absorptive Capacity, and Innovation Performance: Evidence from the Pharmaceutical Industry
Source: PLoS One. 2015 Jul 16;10(7):e0131642. doi: 10.1371/journal.pone.0131642 (PMC4504511; doi:10.1371/journal.pone.0131642)
Supplement: S2 Appendix — This file also contains Table A and Table B. Table A, Unit root test in large pharmaceutical firms. Table B, Unit root test in small and medium pharmaceutical firms. (DOCX) [file pone.0131642.s002.docx]

**S2 Appendix. Unit root test**

**Table A Unit root test in large pharmaceutical firms**

| **Variable** | **AC** | **RD** | **PT** | |
| --- | --- | --- | --- | --- |
|  |  |  | **Level** | **1^st^-difference** |
| **Levin, Lin, and Chu** | -5.32484* | -5.45749* | 1.144 | -9.757* |
| **ADF-Fisher Chi-square** | 123.523* | 96.8587* | 57.452 | 225.070* |
| **PP-Fisher Chi-square** | 140.108* | 124.585* | 55.269 | 486.395* |

AC, Acquisition of external knowledge; RD, R&D intensity; PT, Patents granted;

Null hypothesis: Unit root assumes common (Levin, Lin, and Chu)/individual (ADF/PP-Fisher chi-square) unit root process.

* indicates significance at the 1% level

**Table B Unit root test in small and medium pharmaceutical firms**

| **Variable** | **AC** | **RD** | **PT** |
| --- | --- | --- | --- |
|  |  |  | **Level** |
| **Levin, Lin, and Chu** | -10.1646* | -11.0088* | -3.9248 |
| **ADF-Fisher Chi-square** | 243.448* | 279.748* | 124.663* |
| **PP-Fisher Chi-square** | 278.2538* | 539.6338 | 178.493* |

AC, Acquisition of external knowledge; RD, R&D intensity; PT, Patents granted;

Null hypothesis: Unit root assumes common (Levin, Lin, and Chu)/individual (ADF/PP-Fisher chi-square) unit root process.

* indicates significance at the 1% level
